# Complete Genome of African Swine Fever Virus Genotype II in Central Italy

**DOI:** 10.1128/mra.01364-22

**Published:** 2023-05-11

**Authors:** Monica Giammarioli, Maurilia Marcacci, Maria Teresa Scicluna, Antonella Cersini, Claudia Torresi, Valentina Curini, Massimo Ancora, Antonio Rinaldi, Marcello Giovanni Sala, Elisabetta Rossi, Cristina Casciari, Michela Pela, Claudia Pellegrini, Carmen Iscaro, Cesare Cammà, Francesco Feliziani

**Affiliations:** a Istituto Zooprofilattico Sperimentale Umbria e Marche Togo Rosati, Perugia, Italy; b Istituto Zooprofilattico Sperimentale dell’Abruzzo e del Molise G. Caporale, Teramo, Italy; c Istituto Zooprofilattico Sperimentale Lazio e Toscana, Rome, Italy; Katholieke Universiteit Leuven

## Abstract

We report here the whole-genome sequence of the African swine fever virus (ASFV) genotype II, strain 20355/RM/2022_Italy, identified in a wild boar in the city of Rome (Lazio region, Italy) in April 2022.

## ANNOUNCEMENT

African swine fever (ASF) is a devastating infectious disease of suids caused by African swine fever virus (ASFV). Currently, ASF distribution extends across more than 50 countries on five continents. In 2007, ASFV genotype II was identified in Georgia and since then it has spread to Eurasian countries in wild boar (Sus scrofa) and domestic pig populations (1).

ASFV is the only member of the family *Asfarviridae*, genus *Asfivirus*, and the genome consists of a single molecule of linear, covalently close-ended, double-stranded DNA (dsDNA) of 170 to 194 kbp characterized by high genetic stability. The sequence termini are present as two flip-flop forms that are inverted and complementary with respect to each other (2). Despite 15 years of epidemic circulation, the prototype strain Georgia 2007 has accumulated only a few mutations, which in most cases have been identified in the noncoding regions (3, 4).

In late December 2021, the first evidence of ASFV genotype II in Italy was reported in Ovada municipality (Alessandria province) (5), and after that, several cases were described in Liguria and Piedmont regions. In May 2022, a wild boar was found dead in Rome municipality (Lazio region). DNA was extracted from spleen using the QIAamp UltraSens virus kit (Qiagen, Hilden, Germany) according to the manufacturer’s recommendation and tested positive for ASFV by a specific real-time PCR test (6). Purified DNA was quantified by the Qubit DNA HS assay kit (Thermo Fisher Scientific, Waltham, MA, USA) and then sent to the National Reference Centre for Whole Genome Sequencing of Microbial Pathogens (GENPAT) for metagenomics analysis.

Library preparation was carried out by Illumina DNA Prep, (M) tagmentation (96 samples), according to the manufacturer’s protocol. Deep sequencing was accomplished on the NextSeq 500 using the NextSeq 500/550 midoutput reagent cartridge v2 (300 cycles) (Illumina Inc., San Diego, CA, USA) and standard 150-bp paired-end reads. After quality check and trimming of raw reads using FastQC v0.11.5 and Trimmomatic v0.36, respectively, host depletion was performed by Bowtie2 (7). Trimmed reads were mapped to the reference ASFV Georgia 2007 sequence (FR682468.2) using the iVar v1.3.1 tool (8). A consensus sequence of 190,590 nucleotides (nt) and a mean GC content of 38.39%, with horizontal coverage of 100% and deep mean coverage of 113×, were obtained and annotated using GATU (9) and UGENE (10). The annotation revealed the presence of 195 open reading frames (ORFs) encoding structural proteins and uncharacterized proteins. The ASFV 20355/RM/2022_Italy strain belongs to ASFV genotype II and is closely related to other ASFV genomes publicly available including the 2802/AL/2022 Italy isolate from Piedmont region (ON108571.3). Compared to the reference ASFV Georgia 2007, two missense mutations in the ASFV G ACD 00210 gene (124C > T, Pro42Ser) and I215L gene (578A > G, Glu193Gly) and one synonymous mutation in the NP1450L gene (927C > T, Ile309Ile) were identified. These mutations are unique and have been described neither in the ASFV isolate of Piedmont region nor in other European and Asiatic strains. In order to understand the viral evolution in Italian regions, it is recommended to sequence and analyze multiple ASFV isolates collected in different areas at different time points as recently described for the ASFV incursion into Germany in 2020 (11). The analysis of full-length ASFV genome sequences will be useful to establish the amplitude of the nucleotide mutations within each single cluster and to support the epidemiological investigations for tracing the origin of the outbreaks.

### Data availability.

This whole-genome shotgun project of sample ASFV 20355/RM/2022_Italy has been deposited in DDBJ/ENA/GenBank (https://www.ncbi.nlm.nih.gov) under the accession no. OP605386.1. Raw next-generation sequencing (NGS) reads have been deposited in SRA under accession no. PRJNA946731 (release date 2023-03-20).
